# SOX10-Nano-Lantern Reporter Human iPS Cells; A Versatile Tool for Neural Crest Research

**DOI:** 10.1371/journal.pone.0170342

**Published:** 2017-01-20

**Authors:** Tomoko Horikiri, Hiromi Ohi, Mitsuaki Shibata, Makoto Ikeya, Morio Ueno, Chie Sotozono, Shigeru Kinoshita, Takahiko Sato

**Affiliations:** 1 Department of Ophthalmology, Kyoto Prefectural University of Medicine, Kyoto, Japan; 2 Department of Biomedical Engineering, Faculty of Life Sciences, Doshisha University, Kyotanabe, Japan; 3 Center for iPS Cell Research and Application, Kyoto University, Kyoto, Japan; 4 Department of Frontier Medical Science and Technology for Ophthalmology, Kyoto Prefectural University of Medicine, Kyoto, Japan; 5 Advanced Biomedical Engineering Research Center, Doshisha University, Kyotanabe, Japan; Instituto Butantan, BRAZIL

## Abstract

The neural crest is a source to produce multipotent neural crest stem cells that have a potential to differentiate into diverse cell types. The transcription factor SOX10 is expressed through early neural crest progenitors and stem cells in vertebrates. Here we report the generation of SOX10-Nano-lantern (NL) reporter human induced pluripotent stem cells (hiPS) by using CRISPR/Cas9 systems, that are beneficial to investigate the generation and maintenance of neural crest progenitor cells. SOX10-NL positive cells are produced transiently from hiPS cells by treatment with TGFβ inhibitor SB431542 and GSK3 inhibitor CHIR99021. We found that all SOX10-NL-positive cells expressed an early neural crest marker NGFR, however SOX10-NL-positive cells purified from differentiated hiPS cells progressively attenuate their NL-expression under proliferation. We therefore attempted to maintain SOX10-NL-positive cells with additional signaling on the plane and sphere culture conditions. These SOX10-NL cells provide us to investigate mass culture with neural crest cells for stem cell research.

## Introduction

The neural crest cell is a unique, transient part of ectodermal derivatives in developing vertebrates and has multi-ability to migrate and differentiate into numerous cells including peripheral neurons, glia, craniofacial cartilage, cornea and so on [1]. Initial neural crest cells are raised at the edge of the neural plate and the non-neural ectoderm. According to the formation of the neural folds, neural crest cells subsequently occur epithelial mesenchymal transition to delaminate from dorsal neural tube and migrate through several pathways to reach target tissues and differentiate into various cell types as above [2–4].

It has been identified that a lot of genes, including FGF, WNT and retinoic acid signaling, are involving to neural crest specification and regulation, especially the transcription factor SOX10 is a key regulator for the neural crest cells because it is specifically expressed in initial neural crest cells and defines the stemness of the neural crest cells [5–7]. *SOX10* mutations have been associated with Waardenburg syndrome and Hirschsprung disease. Their defects are recapitulated in *Sox10* heterozygous mice which are viable however display hypopigmentation and aganglionic megacolon [8].

In this study, we focused on the purification and the maintenance of neural crest cells differentiated from human induced pluripotent stem (hiPS) cells with Nano-lantern (NL) knock-in reporter, which is a chimeric fluorescent protein of enhanced Renilla luciferase and Venus [9]. In contrast to the previous SOX10-reporter lines as *SOX10* heterozygous or transgenic cells [8, 10–12], our construct achieved bicistronic expression of NL and targeted *SOX10* gene. We have identified additional proper signaling regulators to maintain SOX10-NL positive cells, although most of NL intensity are not detectable after *in vitro* culture for neural crest cells. SOX10-NL hiPS cells would be used for the research of human neural crest development and neural crest stem cell.

## Materials and Methods

### Ethical statement

This study was carried out according to the regulations of Kyoto Prefectural University of Medicine. The experimental protocols dealing with human subjects were approved by the Ethics committee and the Gene Recombination Experiment Safety Committee of Kyoto Prefectural University of Medicine (permit number: 26–5). Written informed consent was provided by each donor.

### Gene targeting with human iPS cells

To construct a human *SOX10* targeting vector, we inserted 2A-Nano-lantern (NL) [9,13] and loxP-pGK-Neo-loxP (floxedNeo) cassette after the stop codon located on exon4 of *hSOX10* to cause bicistronic expressions of hSOX10 and NL (S1 Fig panel A). The sequence of 2A peptide was produced by synthesized oligos and NL fragments was amplified by PCR with KOD-Plus-Neo polymerase (TOYOBO) and pcDNA3-Nano-lantern (Addgene #51970) to construct pBS-2A-NL-pA. The fragment of floxedNeo was amplified by PCR from pBS-floxedNeo vector [14]. Both of 2A-NL-pA and floxedNeo fragments were ligated into pUC19 vector with In-Fusion HD Cloning Kit (Takara) by manufacture’s protocol (S1 Fig panel B).

For 5’ and 3’ arm of *hSOX10* genomic sequences, they are amplified by PCR with genome DNA extracted from 201B7 hiPS cells [15]. These three fragments, 2A-NL-floxedNeo (2A-NL-fNeo), 5’of hSOX10, and 3’ of hSOX10 were connected with In-Fusion HD Cloning Kit into pDT-A vector (pDT-A-hSOX10arm-2A-NL-fNeo, S1 Fig panel C) [16]. All PCR primers are listed in S1 Table.

To introduce into cultured cells with hSOX10-targeting plasmid vectors, pX330-hSOX10ex4, which was constructed with pX330 vector (Addgene #42230) [17] by ligating oligos into it, and linearized pDT-A-hSOX10arm-2A-NL-fNeo, the electroporator NEPA21 (NEPAGENE) was used for introducing plasmid DNAs into hiPS cells as described [18].

### Cell culture and neural crest differentiation

hiPS cells were maintained on SNL feeder cells, treated with 10 mg/ml of mitomycin (Sigma) in DMEM supplemented with 10% of fetal bovine serum (GIBCO), in Primate ES cell medium (ReproCELL) supplemented with 4 ng/ml recombinant human FGF2 (WAKO) or 100 μg/ml G418 (Nacalai Tesque). Generated SOX10-NL iPS cells were maintained on iMatrix-511 (Nippi) coated plates with StemFit AK03N (Ajinomoto) as described under feeder-free culture system [19].

For neural crest induction, Fukuta *et al*. showed the detailed protocol to induce neural crest cells with hiPS cells [20]. In brief, single SOX10-NL iPS cell was expanded in mTeSR1 medium (STEMCELL Technology) for a few days, and induced into neural crest cells in IMDM and F-12 medium supplemented with chemically defined lipid concentrate (GIBCO), 1% of BSA (WAKO), Insulin-Transferrin-Selenium (GIBCO), and 1-thioglycerol (Sigma). Cultured plates were coated with Geltrex Matrix (GIBCO) or fibronectin (Millipore). SB431542 (Stemgent), CHIR99021 (Stemgent), EGF (WAKO), FGF2 (WAKO) were used for the induction and maintenance of neural crest cells. Embryoid bodies were formed as described to determine the differentiation ability of SOX10-NL hiPS cells [15].

Collected NL-positive neural crest cells were transferred onto fibronectin-coated dishes or Ultra-Low attachment 96well plates (Corning) for sphere formation with neural crest induction medium as describes in the result section.

### Cell sorting

Cultured cells were dissociated with TrypLE select (GIBCO) at 37°C for 5 min for detecting SOX10-NL expression. They were incubated sequentially with antibody against human CD271 (NGFR, p75NTR) conjugates with Alexa647 (BD; diluted 1/100) for 30 min on ice [21]. Dissociated cells were resuspended with 1% bovine serum albumin in PBS. Cell debris was eliminated with a cell strainer (BD; 35 μm) and suspensions were stained with SYTOX Red stain (Molecular Probes) to exclude dead cells. Cells were analyzed and collected by cell sorter using FACSJazz (BD).

### Time-lapse imaging analyses

SOX10-NL positive (and NGFR-positive) and SOX10-NL negative (and NGFR-negative) cells were sorted and seeded onto fibronectin-coated tissue culture dishes for 24 hours. After cells were attached, time-lapse phase contrast images (magnification 10x, 5x5 tiles) were taken at 10 min intervals for 24 hours using BioStation CT (Nikon) at 37°C with 5% CO_2_. Cell migration rate was measured by tracking analysis using CL-Quant software (Nikon) for initial 12 hours with time-lapse images.

### Quantitative PCR analyses

Total RNAs from sorted or cultured cells were extracted using RNeasy micro kit (QIAGEN) and genome DNAs were extracted by PureLink Genomic DNA kits (Invitrogen). For quantitative PCR analyses, single strand cDNA was prepared using SuperScript VILO kit (Invitrogen) as manufacture protocol. All RT-qPCR reactions were carried out in triplicate using THUNDERBIRD SYBR qPCR Mix (TOYOBO), normalized to mRNA level of ribosomal protein L13A (RPL13A) or genome DNA expression level of GAPDH as a control [22]. Primers sequences (5’ to 3’) are listed in S2 Table.

### Immunofluorescence assay

Cultured cells were fixed in 4% paraformaldehyde for 10 min at 4°C, permeabilized with 0.2% Triton and 50 mM NH_4_Cl. Fixed samples were pre-treated with BlockingOne (Nacalai Tesque) for 30 min at RT, and incubated with anti-OCT3/4, anti-NANOG, anti-SSEA4, anti-TRA1-60 (Cell Signaling Technology; diluted 1/200), anti-GFP (Molecular Probes; diluted 1/500), anti-SOX10 (SantaCruz Biotechnology; diluted 1/100) antibodies in 5% of BlockingOne in PBST for overnight at 4°C. After three washes with 0.1% Tween20 in PBS, cells were incubated with Alexa488 or Alexa594-conjugated secondary antibodies (Molecular Probes; diluted 1/500). Cells were washed and mounted in SlowFade Diamond antifade mountant with DAPI (Molecular Probes). Fluorescent images were collected on the software of BZ-X700 (Keyence). For quantitation of cultured cells, numbers of dishes were analyzed from triplicate experiments.

## Results

### Human *SOX10* genomic DNA digestion with CRISPR/Cas9 system

**Fig 1:**
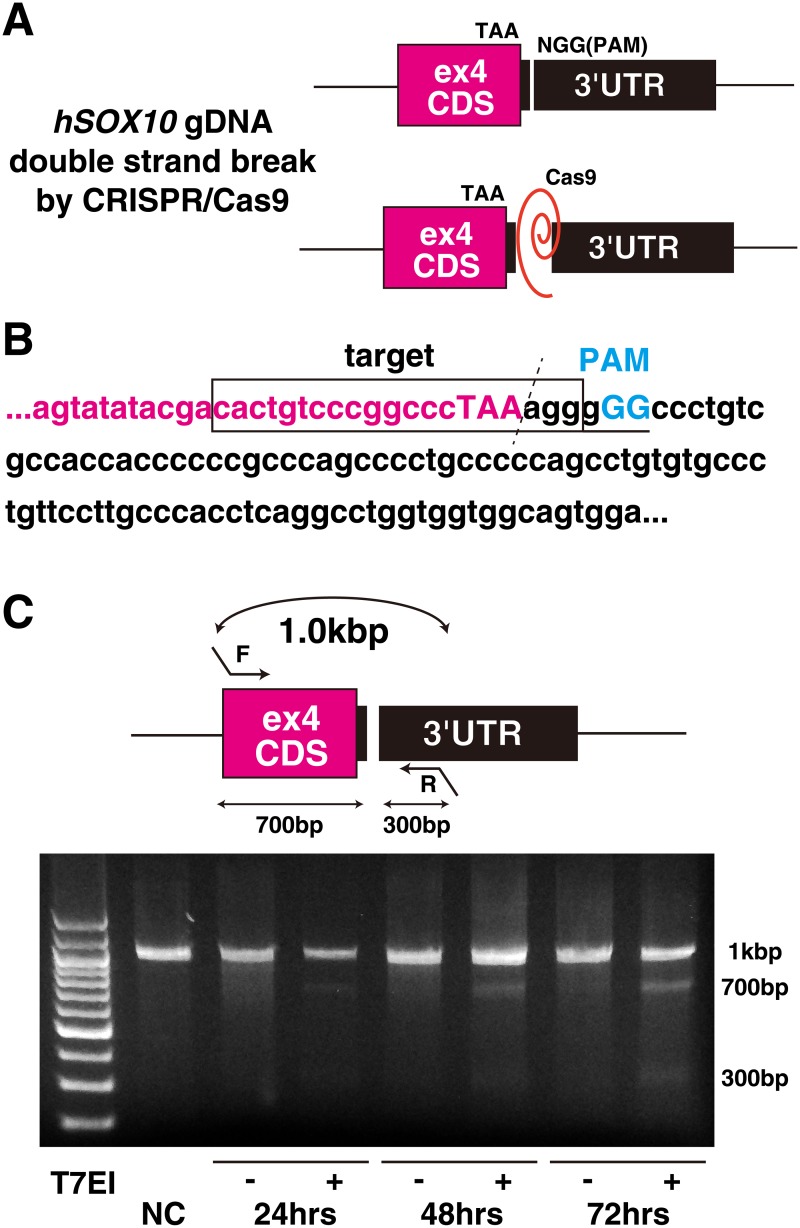
Effect of guide sequence to human *SOX10* on Cas9 activity. (A) A model of Cas9 activity to the human *SOX10* exon4 locus. (B) Cas9 with matching single guide RNA (sgRNA, red) and proto-spacer adaptor motif (PAM, blue) sequences adjacent to human *SOX10* stop codon. (C) T7 endonuclease 1 assay for Cas9-mediated cleavage (700bp and 300bp) on the gel showing comparable modification of targeted human *SOX10* genomic fragment in HEK293T cells according to transfected periods (24 hours, 48 hours, and 72 hours).

To cause double strand break to the border between CDS and 3’UTR in *hSOX10* exon4 (Fig 1A), a Candidate sequence of the guide RNA (cactgtcccggccctaaagg-gGG, hSOX10ex4) to target by Cas9 was selected by CRISPRdirect website (http://crispr.dbcls.jp) [23], and inserted into pX330 vector to create pX330-hSOX10ex4 editing vector (Fig 1B). The effect of double strand break into *hSOX10* genomic sequence of hiPS cells was evaluated by the digestion of heteroduplex PCR products, involving the target region by pX330-hSOX10ex4 editing vector monitored with T7 endonuclease I (T7EI). Digested bands of 700 bp and 300 bp were found in T7EI-treated genome DNA samples, according to treated periods (Fig 1C). The data suggested that Cas9 and the guide-RNA expressing construct has an efficiency to target *hSOX10* genomic sequence.

### Generation of SOX10-NL hiPS reporter line

**Fig 2:**
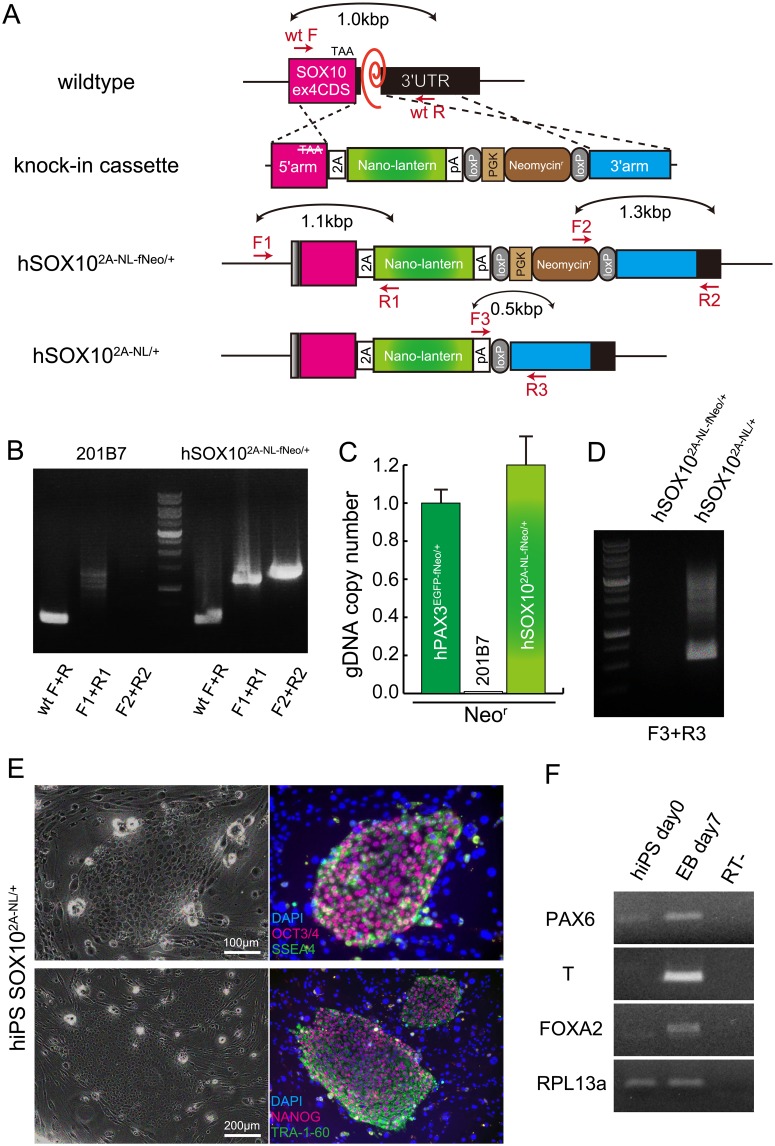
Generation of hSOX10-2A-Nano-lantern reporter hiPS cells. (A) A schematic representation of targeting cassette for *hSOX10*^*2A-NL-fNeo/+*^ or *hSOX10*^*2A-NL/+*^ knockin allele. (B) Genotyping PCR with genome DNA extracted from hiPS 201B7 and *hSOX10*^*2A-NL-fNeo/+*^ hiPS cells. Genomic recombined 5’fragment of targeting cassette was detectable by F1 and R1 primers, and also 3’ fragment by F2 and R2 primers. (C) DNA Copy number check by quantitative PCR with genome DNA extracted from hiPS 201B7, *Pax3*^*EGFP-fNeo/+*^, and *hSOX10*^*2A-NL-fNeo/+*^ cells. All error bars indicate ±s.e.m. (n = 3) (D) The excision of loxP-Neo-loxP genomic fragment in *hSOX10*^*2A-NL-fNeo/+*^ hiPS cells with Cre recombinase treatment to establish *hSOX10*^*2A-NL/+*^ hiPS cells. (E) *hSOX10*^*2A-NL-fNeo/+*^ cells were immunostained with indicated undifferentiated pluripotent cell markers, anti-OCT3/4 (red, upper panel) and anti-SSEA4 (green, upper panel), anti-NANOG (red, lower panel) and anti-TRA1-60 (green, lower panel) antibodies. Nuclei were stained with 4’6-diamidino-2-phenylindole (DAPI, blue). Scale bar, 100 μm. (F) Differentiation capacity into ectoderm (*PAX6*), mesoderm (*T*), endoderm (*FOXA2*) markers with embryonic bodies (EB day7) from *hSOX10*^*2A-NL/+*^cells.

In order to construct the targeting vector for human *SOX10* as designed in S1 Fig, the targeting sequences along with 5’ and 3’ homology arms (0.7 kbp and 1.0 kbp, respectively) were amplified by PCR and combined with 2A-NL-floxedNeo reporter sequence by In-Fusion technology efficiently into pDT-A-hSOX10arm-2A-NL-fNeo vector (Fig 2A, knock-in cassette).

hiPS cells were electroporated with pDT-A-hSOX10arm-2A-NL-fNeo targeting vector and pX330-hSOX10ex4 editing vector. The electroporated cells were plated on SNL feeder cells, and G418 selection was started two days later for a week. Single colonies were picked up, expanded and subsequently genomic DNA was used for screening the correct insertion of knock-in reporter cassette. PCR results showed that selected clone contained reporter cassette (Fig 2B, F1+R1, F2+R2). And this clone has been confirmed to obtain one copy of the targeted Neomycin resistance gene allele by comparing with known knock-in hiPS cell (Fig 2C, *hSOX10*^*2A-NL-fNeo/+*^). To excise the Neomycin resistance gene cassette from *hSOX10*^*2A-NL-fNeo/+*^ hiPS cells, pCAG-Cre vector was transiently transfected [24], and single hiPS colonies were picked up and confirmed the lack of floxed Neo sequence by PCR (Fig 2D, *hSOX10*^*2A-NL/+*^).

The selected hiPS clone *hSOX10*^*2A-NL/+*^ was confirmed to express pluripotency markers with OCT3/4, NANOG, SSEA4 and TRA-1-60, after gene targeting and antibiotic selection experiments (Fig 2E). This clone also had the multipotency to differentiate into ectodermal (*PAX6*), mesodermal (*T*), and endodermal (*FOXA2*) cells in embryoid bodies (Fig 2F). This clone was therefore chosen for the induction experiments to neural crest cells.

### Confirmation of SOX10-NL activity during neural crest differentiation

**Fig 3:**
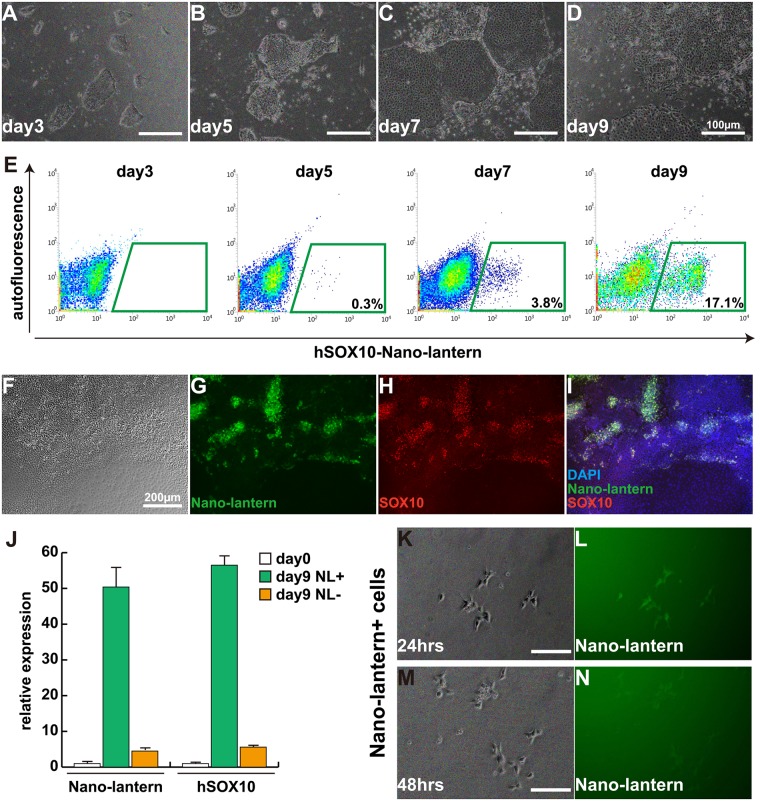
Induction of Nano-lantern-positive cells from *hSOX10*^*2A-NL/+*^ hiPS cells. (A-D) Morphology of cultured cells from *hSOX10*^*2A-NL/+*^ hiPS cells during 3 days (Day3) to 9 days (Day9). Scale bar, 100 μm. (E) FACs profiles for detecting Nano-lantern-positive cells from induced cells. (F-I) cultured cells for 7 days were immunostained with anti-GFP (Nano-lantern; G, green) and anti-SOX10 (H, red) antibodies. Nuclei were stained with 4’6-diamidino-2-phenylindole (DAPI, blue). Scale bar, 200 μm. (J) Relative expressions of *Nano-lantern* and *hSOX10* with Nano-lantern-positive (NL+) or negative (NL-) cells by FACs analyses. All error bars indicate ±s.e.m. (n = 3) (K-N) Cultured Nano-lantern-positive cells decrease the fluorescent intensity of Nano-lantern expression after 24 hours (K, L) to 48 hours (M, N). Scale bar, 50 μm.

hiPS *SOX10*^*2A-NL/+*^ reporter cells were differentiated into the neural crest lineage using the protocol as described [20], with SB431542, a specific inhibitor of TGFß receptor kinase, and CHIR99021, an inhibitor of GSK3 to function as a WNT activator, for nine days (Fig 3A–3D), and NL activity and SOX10 expression were analyzed during the differentiated periods. Analyses of Fluorescence activated cell sorting (FACS) and luciferase activity showed NL reporter activity started to increase about day 5 of differentiation and around 20% of NL positive (NL+) cells were detected at day 9 of differentiation (Fig 3E, and S2 Fig). These differentiated cells at day 7 were fixed and stained with anti-SOX10 antibody to confirm co-expression of NL and SOX10. As expected, NL+ cells co-express SOX10, indicating that NL-reporter cassette mimics the activation of endogenous SOX10 expression (Fig 3F–3I). We also confirmed the co-expression by detecting enriched *SOX10* transcripts in NL+ sorted cells (Fig 3J). In order to expand NL+ sorted cells, NL+ cells were grown in described culture media supplemented with 10 ng/mL of FGF2 and 10 ng/mL of EGF on fibronectin-coated plates^16^. NL fluorescent expression could be detected in NL+ sorted cells cultured for 24 hours under microscopy, however their NL intensity was attenuated in cultured cells for 48 hours (Fig 3K–3N).

### Characteristics of SOX10-NL+ neural crest cells

**Fig 4:**
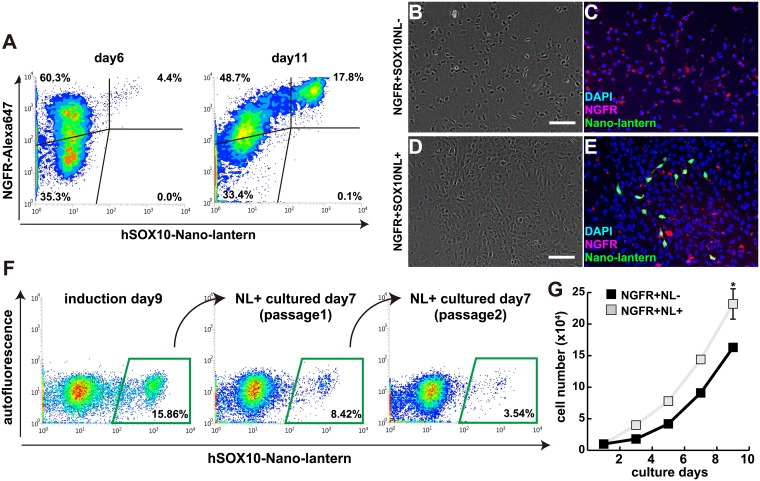
Characteristics of Nano-lantern-positive neural crest cells. (A) Flow cytometry plots of induced Nano-lantern-positive cells from *hSOX10*^*2A-NL/+*^ hiPS cells for 6 days (day 6) or 11 days (day 11). All Nano-lantern-positive cells indicate in NGFR (p75)-positive fraction. (B-E) FACs-purified cells (NGFR+NL-; B, C or NGFR+NL+; D, E) were cultured for 7 days and immunostainted with anti-NGFR (red in C, E) and anti-GFP (Nano-lantern; green in C, E) antibodies. Nuclei were stained with 4’6-diamidino-2-phenylindole (DAPI, blue). Scale bar, 100 μm (F) Continuously decreased Nano-lantern-positive percentages in Nano-lantern-positively sorted and cultured cells. (G) Nano-lantern-positive cells (NGFR+NL+) grow faster than NGFR-positive; Nano-lantern-negative cells (NGFR+NL-). All error bars indicate ±s.e.m. (n = 3). *P* values are determined by t-test from a two-tailed distribution. *P<0.05.

SOX10-NL+ cells were purified by FACS as neural crest cells differentiated from hiPS cells. The SOX10-NL+ population isolated from differentiated neural crest cells for day 6 to day 11 exhibited all co-expression with NGFR (Fig 4A), indicating that NL+ population was identified with neural crest marker NGFR. However the activity of NL was not detectable in all NGFR+ population (Fig 4A). To assess the identity of the NGFR+; NL+ or NGFR+; NL-negative (NGFR+NL-) cells, we investigated their differences. Same numbers of NGFR+NL+ or NGFR+NL- sorted cells were seeded on fibronectin-coated dishes and expanded for a week. Almost cultured cells were detected with NGFR+ in both conditions (Fig 4B–4E), however the percentage of NL+ was decreased in this culture condition as shown in Fig 3 (Fig 4E). And this NL+ proportion was declined according to passage numbers (Fig 4F). The cell numbers after expansion of NL+ cells showed much higher than those of NGFR+NL- cells, indicating that NL+ neural crest cells have a competent to be propagated than NGFR+ neural crest cells (Fig 4G).

### Effects of pivotal exogenous factors to maintain SOX10-NL positive neural crest cells

**Fig 5:**
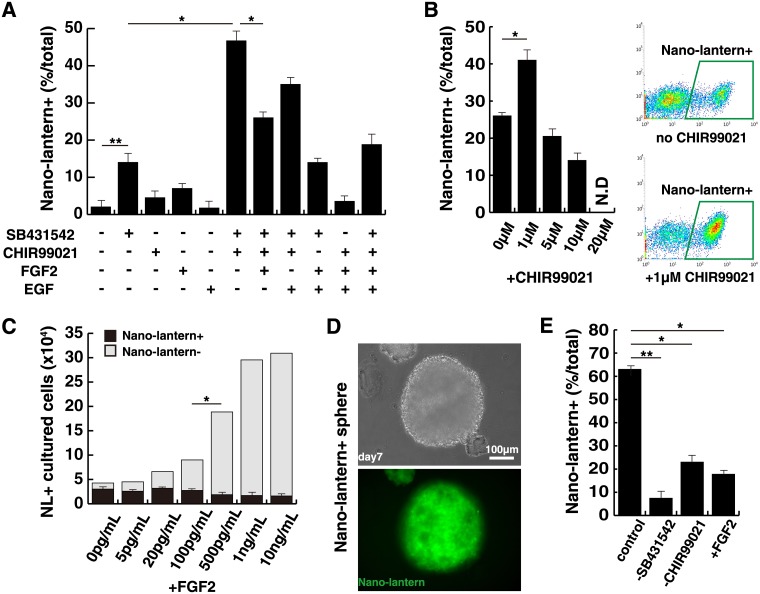
effects of exogenous factors to SOX10-Nano-lantern positive cells. (A) The combination effect of SB431542 and CHIR99021 inhibitors, FGF2, and EGF to cultured Nano-lantern-positive cells. (B) Different CHIR99021 concentrations for the expansion of Nano-lantern-positive cells. Cultured Nano-lantern-positive cells were analyzed by FACs with or without CHIR99021 (right panels). (C) Effects of FGF2 for the expansion of Nano-lantern-positive cells. total cultured cells were counted and the percentage of Nano-lantern-positive cells were analyzed. (D) 7 days cultured images of aggregated spheres derived from Nano-lantern+ cells (below; NL fluorescence image) (E) Effects to NL+ proportion in aggregated spheres without SB431542 and CHIR99021, or with FGF2. All error bars indicate ±s.e.m. (n = 3). *P* values are determined by t-test from a two-tailed distribution. *P<0.05; **P<0.01.

SOX10-NL+ cells had a differentiation potential into cells of peripheral nervous system, and mesenchymal stem cells (S3 Fig), and an ability of cell migration (S4 Fig and S1 Movie), however these cells do not remain the activity of NL but rather spontaneously differentiate into NGFR+NL- cells in culture medium containing SB431542, FGF2 and EGF. We therefore sought the appropriated condition to expand cells keeping the NL activity. Subsequent screening of inhibitors and growth factors revealed that SB431542 was essential for the increase of the ratio of NL+ cells (Fig 5A). CHIR99021 also had the effect to NL+ cell positively, however high dose of this inhibitor (higher than 5 μM) conversely down-regulated the ratio of NL+ cells (Fig 5B). As shown in Fig 5A, we found that FGF2 might have the negative effect to NL+ activity although cultured cells supplemented with 10 ng/mL of FGF2 were grown well and healthy because of few dead cells, which were stained with SYTOX red (not shown). However high doses of FGF2, more than 500 pg/mL drastically reduced the NL activity, and the proportion of NL+ in NL+ cultured cells was gradually diminished by the amount of FGF2 (Fig 5C).

Kerosuo *et al*. recently indicated epithelial sphere culture of premigratory neural crest cells to maintain their self-renewing and multipotent state [25]. We tested to determine optimal conditions for enabling SOX10-NL+ cells to aggregate. Spheres with NL+ cells were successively formed in the same condition of monolayer culture of NL+ cells, with 10 μM SB431542 and 1 μM CHIR99021 (Fig 5D). Furthermore, the proportion of NL+ cells in sphere culture condition was significantly reduced without SB431542 or CHIR99021, or with FGF2 (Fig 5E), compatible with the monolayer culture (Fig 5A). These data suggested that these pivotal factors in TGFß, WNT and FGF signaling are effective for the maintenance of NL positively regardless of cell-cell interaction.

## Discussion

Here we present successful generation of SOX10-NL knock-in hiPS cells by the CRISPR/Cas9 and In-Fusion technologies, which are valuable to monitor SOX10 expression by both of fluorescent and luciferase activities. Nano-lantern enable us to perform not only real-time imaging in living cells as other fluorescent proteins, but also in vivo imaging by sensitive luminescent detection. This SOX10-NL hiPS cell lines can be also used for evaluation of directed differentiation toward neural crest lineage using chemical screening as well as to study temporal emergence of neural crest stem cells during *in vitro* differentiation. Moreover, our approach shown here is simple, efficient and versatile to be applicable to generate other reporter knock-in cells, and ideal gene targeting for predominant genotype like *SOX10* because Haploinsufficiency of hetelozygous *SOX10* causes pigmentation and megacolon defect in Hirschsprung disease [8]. Therefore, we chose not to generate *SOX10* heterozygotes or BAC transgenic cells, which might affect to intact *SOX10* or other gene expression, but to target 3’UTR sequence of *SOX10* just with sequences of 2A signal peptide and Nano-lantern instead of *SOX10* stop codon to function intact *SOX10* coding sequence.

Although a variety of neural crest-derived cells, differentiated from the pluripotent stem cells have been used for each experiment, it has difficulty to maintain premigratory embryonic neural crest progenitors or neural crest stem cells marked by SOX10, without spontaneous differentiation in vitro. We found that it was possible to induce SOX10-positive neural crest cells derived from hiPS cells as shown induction method before [20], although these cells were immediately differentiated into the NGFR-positive but SOX10-negative population because of additional FGF signaling, which support their growth. So we identified the necessity of the inhibition of TGFß, and the appropriate concentration of CHIR99021 and FGF2 for maintaining SOX10-NL activity in vitro culture. And this condition for SOX10-maintaining cell culture system also adapted to neural crest-sphere culture as well.

Taken together, these results demonstrate that SOX10-expressing cells have self-renewal capacity and a possibility to form neural crest derivatives under proper differentiated conditions, and provide mass culture method of neural crest cells and would promote the research to use derivatives from human neural crest cells for cell therapy or drug screenings.

## Supporting Information

S1 Fig General strategy of 2A-Nano-lantern (NL) targeting into the human *SOX10* allele. (A) Schematic diagram of the human *SOX10* locus on chromosome 22 and targeting cassette for SOX10-2A-NL-floxed Neo (*hSOX10*^*2A-NL-fNeo/+*^) or SOX10-2A-NL (*hSOX10*^*2A-NL/+*^) knockin allele with genomic double strand break caused by CRISPR/Cas9 system. (B) PCR-amplified 2A-NL and loxP-pGK-Neo-loxP fragments were recombined with In-fusion clonase into pUC19 vector (pUC19-2A-NLpA-floxedNeo). (C) Triple DNA fragments (*hSOX10* 5’arm, *hSOX*10 3’arm and 2A-NL-floxedNeo) were recombined with In-fusion clonase into pDT-A vector for targeting into the human *SOX10* allele.
(PDF)

S2 Fig Luciferase activities of Nano-lantern with induced neural crest cells from *hSOX10*^*2A-NL/+*^ hiPS cells. Collected cells were counted and analyzed with Renilla Luciferase Assay System (Promega) as manufacturing protocol.
(PDF)

S3 Fig Induced cells of the peripheral nervous system (PNS) and mesenchymal stem cells differentiated from SOX10-Nano-lantern positive neural crest cells. (A) NL+ cells were differentiated for 14 days supplemented with N2 supplement (GIBCO), 10 ng/mL of BDNF, GDNF, NT-3 and NGF (WAKO). Differentiated cells were immunostained with anti-ß3-tubulin (right panel). Nuclei were stained with 40,6-diamidino-2-phenylindole (DAPI, middle panel). Scale bar, 100 μm. (B) Expression of cell surface markers in mesenchymal stem cells.
(PDF)

S4 Fig SOX10-Nano-lantern positive cells migrate to appropriate chemoattractants. (A) Representative migrated images from the colony of confluent SOX10-NL+ cells after 36 hours with or without chemoattractants. (B) NL+ cells migrated to chemoattractants with BMP2, FGF8 and SDF1. (C) Sorted NL+NGFR+ cells displayed higher migration rate than NL-NGFR- cells as shown in S1 Movie. *P<0.05, **P<0.01.
(PDF)

S1 Movie Movie data for tracking analysis of SOX10-Nano-lantern positive cells. SOX10-NL+NGFR+ cells (left panel) and SOX10-NL-NGFR- cells (right panel).
(WMV)

S1 Table primer sequences for *SOX10-2A-Nanolantern* targeting vector. (PDF)

S2 Table Primer sequences for RT-PCR or genomic PCR used in this study. (PDF)
