# Supplementary figures and images for: SOX10-Nano-Lantern Reporter Human iPS Cells; A Versatile Tool for Neural Crest Research
Source: PLoS One. 2017 Jan 20;12(1):e0170342. doi: 10.1371/journal.pone.0170342 (PMC5249153; doi:10.1371/journal.pone.0170342)

S1 Fig

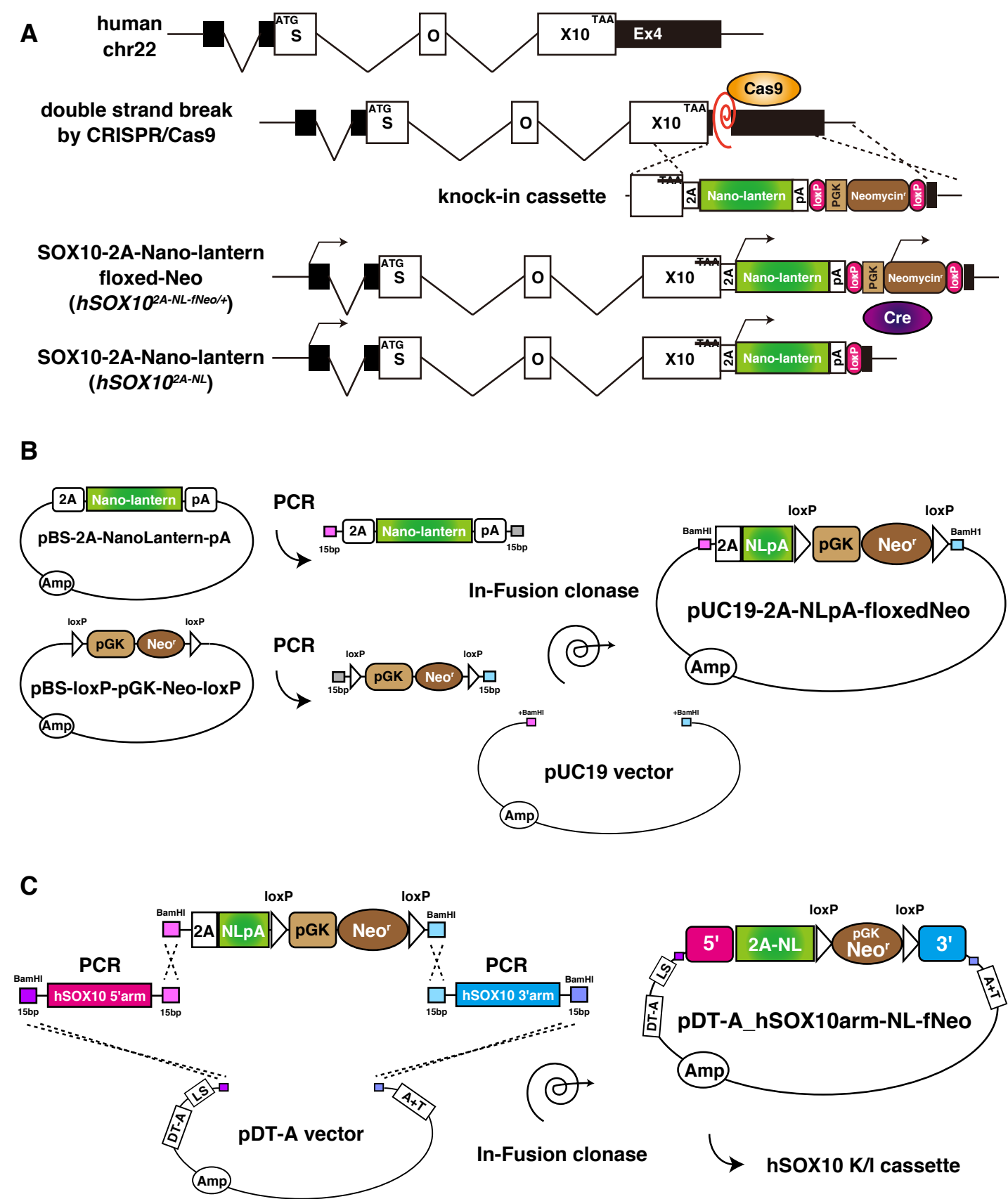

Supplement: S1 Fig — (A) Schematic diagram of the human SOX10 locus on chromosome 22 and targeting cassette for SOX10-2A-NL-floxed Neo (hSOX102A-NL-fNeo/+) or SOX10-2A-NL (hSOX102A-NL/+) knockin allele with genomic double strand break caused by CRISPR/Cas9 system. (B) PCR-amplified 2A-NL and loxP-pGK-Neo-loxP fragments were recombined with In-fusion clonase into pUC19 vector (pUC19-2A-NLpA-floxedNeo). (C) Triple DNA fragments (hSOX10 5’arm, hSOX10 3’arm and 2A-NL-floxedNeo) were recombined with In-fusion clonase into pDT-A vector for targeting into the human SOX10 allele. (PDF) [file pone.0170342.s001.pdf]

S2 Fig

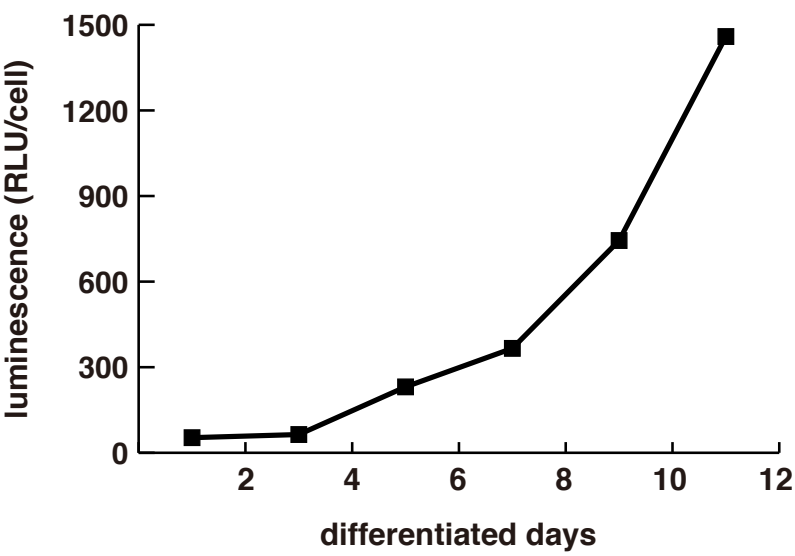

Supplement: S2 Fig — Collected cells were counted and analyzed with Renilla Luciferase Assay System (Promega) as manufacturing protocol. (PDF) [file pone.0170342.s002.pdf]

S3 Fig

A

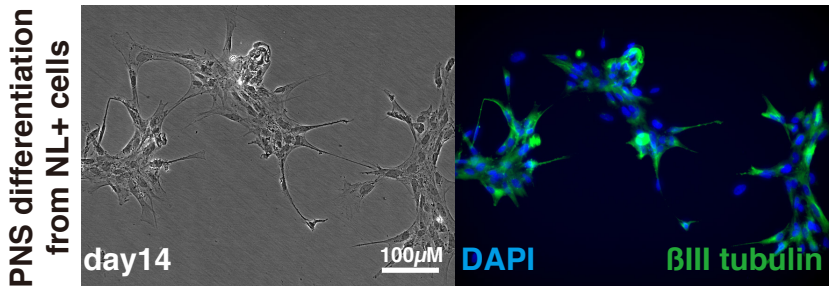

B

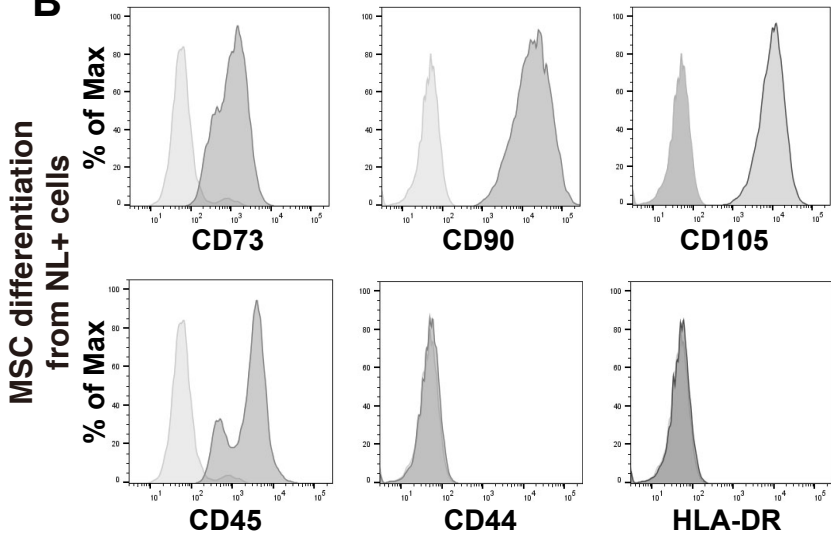

Supplement: S3 Fig — (A) NL+ cells were differentiated for 14 days supplemented with N2 supplement (GIBCO), 10 ng/mL of BDNF, GDNF, NT-3 and NGF (WAKO). Differentiated cells were immunostained with anti-ß3-tubulin (right panel). Nuclei were stained with 40,6-diamidino-2-phenylindole (DAPI, middle panel). Scale bar, 100 μm. (B) Expression of cell surface markers in mesenchymal stem cells. (PDF) [file pone.0170342.s003.pdf]

S4 Fig

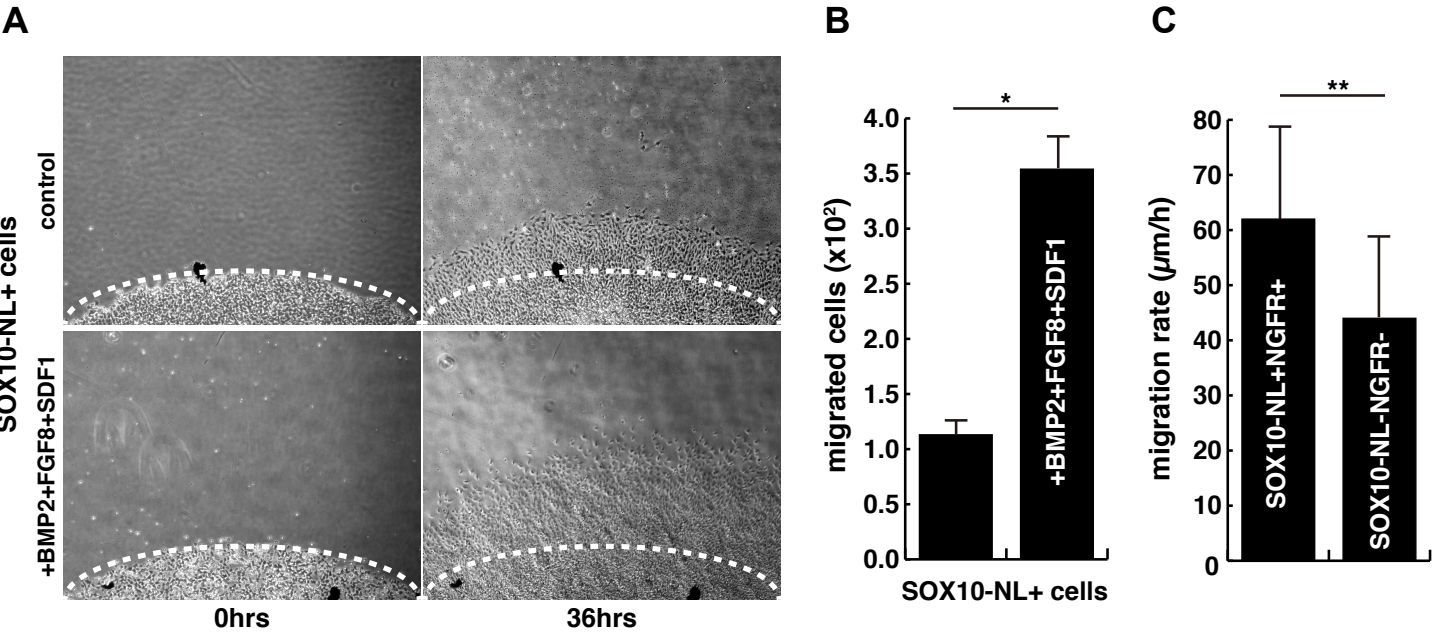

Supplement: S4 Fig — (A) Representative migrated images from the colony of confluent SOX10-NL+ cells after 36 hours with or without chemoattractants. (B) NL+ cells migrated to chemoattractants with BMP2, FGF8 and SDF1. (C) Sorted NL+NGFR+ cells displayed higher migration rate than NL-NGFR- cells as shown in S1 Movie. *P<0.05, **P<0.01. (PDF) [file pone.0170342.s004.pdf]
