# Supplementary material for: SOX10-Nano-Lantern Reporter Human iPS Cells; A Versatile Tool for Neural Crest Research
Source: PLoS One. 2017 Jan 20;12(1):e0170342. doi: 10.1371/journal.pone.0170342 (PMC5249153; doi:10.1371/journal.pone.0170342)
Supplement: S1 Table — (PDF) [file pone.0170342.s006.pdf]

Primer name	Sequence (5' to 3')	Comments
hSOX10ex4 guide_F	cacccactgtcccggccctaaagg	pX330 cloning (Fig.1)
hSOX10ex4 guide_R	aaacccctttagggccgggacagtg	
pUC19_2A_F	cggtacccggggatccggaagcggagctac	2A-*Nanolantern* fragment (S1 Fig panel B)
pA_fNeo_In-fusion_R	gcggccgc tccccagcatgcctgctattgt	
pA_fNeo_In-fusion_F	ctggggagcggccgcaattagtgataactt	floxedNeo fragment (S1 Fig panel B)
fNeo_pUC19_R	cgactctagaggatccgagctcagacgata	
hSOX10ex4 5'arm_F	tgtaattccagctactcaggaggctgaggc	5'arm (704bp, S1 Fig panel C)
hSOX10ex4 5'arm_R	gccagagccatggcccacccacccct	
hSOX10ex4 3'arm_F	cagcccctgcccccagcctgtgtgccctgt	3'arm (1103bp, S1 Fig panel C)
hSOX10ex4 3'arm_R	ggggctagagtggctaggagaggggactac	
pDT-A_Xho1_A+T_F	cactggccgtcgttttacctcgagagtaat	pDT-A vector (S1 Fig panel C)
pDT-A_linearlized_R	atttaaatgcggccgcgtttaaacggccgg	
