# Supplementary material for: SOX10-Nano-Lantern Reporter Human iPS Cells; A Versatile Tool for Neural Crest Research
Source: PLoS One. 2017 Jan 20;12(1):e0170342. doi: 10.1371/journal.pone.0170342 (PMC5249153; doi:10.1371/journal.pone.0170342)
Supplement: S2 Table — (PDF) [file pone.0170342.s007.pdf]

target	Forward (5'to 3')	Reverse (5' to 3')	product size
SOX10genomic exon4 WT (Fig.1C, Fig.2B)	gccagagccatggcccacccacccctccaa	tgggatagagggtcattcctgggggaaggt	1kbp
SOX10-2A-NL F1+R1 (Fig.2B)	ggttttcccagaggcacccgc	ggacttgaagaagtcgtgctg	1148bp
SOX10-2A-NL F2+R2 (Fig.2B)	cttttctggattcatcgactgtg	ccactgggaggcaaggggtaag	1310bp
Neo (Fig.2C)	cctgaatgaactgcaggacg	cttcccgcttcagtgacaac	99bp
DLX5 (Fig.2C)	ttccaagctccgttccagac	ccccgtagggctgtagtagt	108bp
SOX10-2A-NL F3+R3 (Fig.2D)	gagctcgctgatcagcctcgact	tgggatagagggtcattcctgggggaaggt	513bp
PAX6 (Fig.2F)	tgtccaacggatgtgtgagt	tttcccaagcaaagatggac	162bp
T (Fig.2F)	gccctctccctcccctccacgcacag	cggcgccgttgctcacagaccacagg	275bp
FOXA2 (Fig.2F)	tgggagcggtgaagatggaagggcac	tcatgccagcgcccacgtacgacgac	216bp
RPL13A (Fig.2F, Fig. 3J)	ccctggaggagaagaggaaa	acgttcttctcggcctgttt	91bp
Nanolantern (Fig.3J)	cgaccactaccagcagaaca	gaactccagcaggaccatgt	133bp
SOX10 (Fig.3J)	cggaccagtacccgcacct	ggcgcttgtcactttcgttca	87bp
